# Genome-Wide Identification of the CBF Gene Family and ICE Transcription Factors in Walnuts and Expression Profiles under Cold Conditions

**DOI:** 10.3390/ijms25010025

**Published:** 2023-12-19

**Authors:** Huijuan Zhou, Jiayu Ma, Hengzhao Liu, Peng Zhao

**Affiliations:** 1 Xi’an Botanical Garden of Shaanxi Province, Institute of Botany of Shaanxi Province, Xi’an 710061, China; ericguanyuzhao@163.com; 2 Key Laboratory of Resource Biology and Biotechnology in Western China, Ministry of Education, College of Life Sciences, Northwest University, Xi’an 710069, China; majiayu@stumail.nwu.edu.cn (J.M.); hengzhaoliu@stumail.nwu.edu.cn (H.L.)

**Keywords:** walnut, CBF, ICE1, low temperature, gene family

## Abstract

Cold stress impacts woody tree growth and perennial production, especially when the temperature rapidly changes in late spring. To address this issue, we conducted the genome-wide identification of two important transcription factors (TFs), CBF (C-repeat binding factors) and ICE (inducers of CBF expression), in three walnut (*Juglans*) genomes. Although the CBF and ICE gene families have been identified in many crops, very little systematic analysis of these genes has been carried out in *J. regia* and *J. sigillata*. In this study, we identified a total of 16 *CBF* and 12 *ICE* genes in three *Juglans* genomes using bioinformatics analysis. Both CBF and ICE had conserved domains, motifs, and gene structures, which suggests that these two TFs were evolutionarily conserved. Most *ICE* genes are located at both ends of the chromosomes. The promoter *cis*-regulatory elements of *CBF* and *ICE* genes are largely involved in light and phytohormone responses. Based on 36 RNA sequencing of leaves from four walnut cultivars (‘Zijing’, ‘Lvling’, ‘Hongren’, and ‘Liao1’) under three temperature conditions (8 °C, 22 °C, and 5 °C) conditions in late spring, we found that the *ICE* genes were expressed more highly than *CBFs*. Both CBF and ICE proteins interacted with cold-related proteins, and many putative miRNAs had interactions with these two TFs. These results determined that CBF1 and ICE1 play important roles in the tolerance of walnut leaves to rapid temperature changes. Our results provide a useful resource on the function of the *CBF* and *ICE* genes related to cold tolerance in walnuts.

## 1. Introduction

Cold stress (low temperature) is an important environmental factor significantly constraining plant growth, development, and geographical distribution [1,2]. Cold stress has effects on crop productivity, such as rice and wheat yield [2]. To acclimate to the environmental temperature changes, plants have evolved a cascade of physiological and biochemical regulatory strategies to avoid the limitations [3,4]. There are two different cold signals, rapid and gradual temperature decreases, referring to a complex gene regulation pathway involving transcriptional upregulation or downregulation. It is crucial that C-repeat binding factors/drought response element binding factor (CBFs/DREBs) pathways are upregulated for cold tolerance [5,6,7]. The CBF pathway responding to cold stress is found in numerous plants, such as *Acer* species [8], soybean [9], *Gossypium* [10], *Malus baccata* [11], tomato [12], and *Betula platyphylla* [13], etc. In plants, the ICE1-CBF-COR transcriptional cascade is the key regulation pathway for cold temperature [14,15]. Furthermore, the *CBFs*/*DREBs* genes are regulated by the inducer of CBF expression (ICEs, inducer of CBF expression) to enhance cold tolerance in plants [16,17,18]. The conserved CRT/DRE elements (C-repeat/drought-responsive elements) are induced by freezing or chilling temperatures, and then they bind to the promoter of *COR* (*Cold Regulated*) genes, leading to the stimulation of the target genes’ activity [2,5]. The CBFs/DREB1s belonging to the AP/ERF (APETALA2/ethylene-responsive factor) superfamily bind to DREs and act as act as master regulators in cold-inducible gene expression [14]. There are four *CBF* genes and two *DDF* genes (Dwarf and delayed flowering) in *Arabidopsis thaliana*: *CBF1/DREB1B*, *CBF2/DREB1C*, *CBF3/DREB1A*, *CBF4/DREB1D*, *DDF1/DREB1E*, and *DDF2/DREB1F*. The *ICE1* gene is the MYC-like basic helix-loop-helix transcription factor (bHLH), and it is recognized as a positive inducer of *CBF/DREB* [19].

*Juglans regia* (2n = 32, Persian walnut), being wind-pollinated and monoecious, is an important woody oil tree cultivated worldwide, naturally distributed in Eurasia and even southeastern Europe [20,21,22]. *J. sigillata* (iron walnut) is the most closely related to Persian walnut, which grows in the southwest of China [23,24,25]. The kernels of Persian walnut and iron walnut are rich in nutrition and high in fatty acid content [20,21,22,23,24,25]. Persian walnut is the most important species in *Juglans*, which is distributed and cultivated widely across the world [21,22]. Persian walnuts and iron walnuts are also used as timber tree species for their high-quality wood [20,21,23]. It is commonly known that the flowering and pollinating time of walnuts is in late spring [26,27]. However, there is a limitation for pollinating quality and walnut yield in temperate regions where temperatures can plunge rapidly in the period of late spring frost (cold shock), especially in northern areas [28,29,30]. This extremely serious climate change can cause damage to the flower buds and even entire plants [31,32,33]. Climate change may affect the growth of woody tree species in their distribution region, as low temperatures affect plant development and growth in wild walnuts [23,30]. The developments in whole genome sequencing in walnut and woody species have revealed evidence of cold and chilling stress [28,31,32,33,34] and the genome-wide identification of gene families related to stress studies in *Juglans* species [35,36,37,38]. CBF and ICE1, as important transcription factors in response to cold stress, may enhance the cold resistance of plants under low-temperature conditions by regulating the essential functional genes [39,40,41]. The cold hardiness of wood plants is related to their productivity, fruiting, and ecosystem functions [31,42]. Therefore, taking into consideration the economic losses in walnut yield due to cold spells, it is important to identify the molecular basis for the cold stress response in the cultivars and varieties of walnuts. However, as the important regulators of the cold stress response in plants, little is known about the CBF and ICE1 gene family in Persian walnuts and iron walnuts so far.

In this study, based on the high-quality genomes of three walnut varieties including *J. regia* (‘Chandler’) [20], *J. regia* (‘Zhongmucha-1’) [43], and *J. sigillata* (‘Yangbi’) [23], we performed a genome-wide identification and systematically characterization of the CBF and ICE1 in three walnut genomes. We also detected the phylogenetic tree, gene structure, motifs, conserved domains, and *Cis*-acting elements in these genomes. We detected the transcriptional changes and the levels of related gene expression in the three different cultivars under different low-temperature conditions in late-spring frost. The results could provide a reference for reducing the effect of late spring frost on walnut production.

## 2. Results

### 2.1. Genome-Wide Identification and Phylogenetic Analysis of the CBF and ICE Gene Family in Three Walnut Genomes

**Figure 1:**
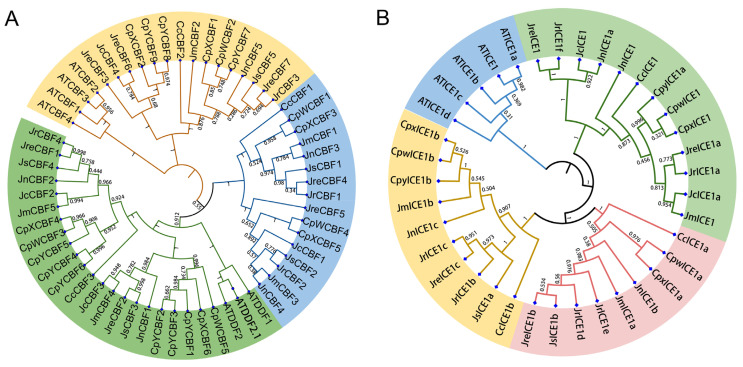
The Neighbor-Joining (NJ) phylogenetic tree of CBF proteins and ICE proteins from *Arabidopsis thaliana*, *Juglans regia* (‘Chandler’) [20], *J. regia* (Zhongmucha-1) [43], *J. sigillata* (iron walnut) [23], *J. mandshurica*, *J. nigra*, and *Cyclocarya paliurus*, respectively. (**A**) The NJ tree of CBF proteins. (**B**) The NJ tree of ICE proteins.

The whole-genome data from three walnut genomes and those from another five species (Table S1) were used to identify the CBF and ICE1 gene family. A total of 16 *CBFs* (4 *JrCBF*, 7 *JreCBF*, and 5 *JsCBF*) and 12 *ICEs* (6 *JrICE*, 4 *JreICE*, and 2 *JsICE*) were identified in the three walnut cultivars, respectively (Table 1). These CBFs were divided into three subgroups (subgroup I, subgroup II, and subgroup III) and four subgroups of *ICE1* genes (subgroup I, subgroup II, subgroup III, and subgroup IV) (Figure 1). The largest subgroup included 25 members of the CBF gene family (green branch, Figure 1A). The results indicated that ATCBFs and ATDDFs were divided into different groups. The largest subgroup included 13 members of the ICE gene family (green branch), followed by another 10 members of the ICE gene family (orange branch, Figure 1B). The subgroup with the most members of the ICE1 gene family in *A. thaliana* was represented by a blue branch (Figure 1B).

### 2.2. Chromosomal Distribution and Duplication Patterns of Juglans regia and Juglans sigillata

**Figure 2:**
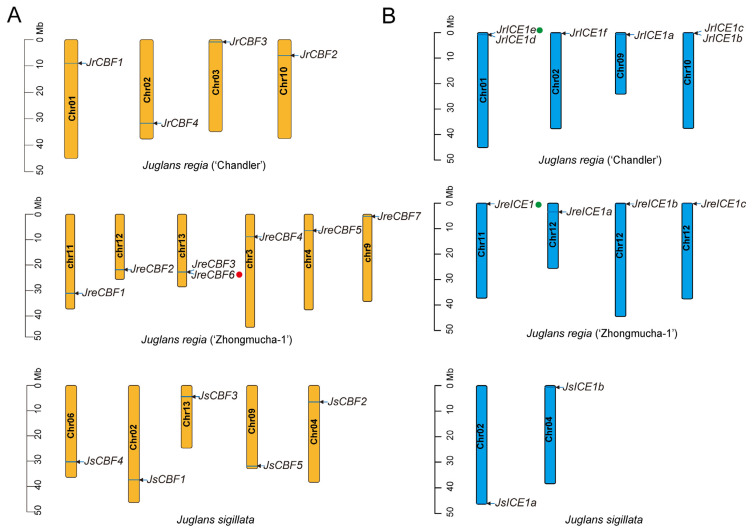
Chromosomal locations of *CBF* genes and *ICE1* genes in *Juglans regia* (‘Chandler’) [20], *J. regia* (‘Zhongmucha-1’) [43], and *J. sigillata* (iron walnut) [23], respectively. (**A**) Chromosomal localization position and duplication types of the *CBF* genes in *Juglans regia* (‘Chandler’) [20], *J. regia* (‘Zhongmucha-1’) [43], and *J. sigillata* (iron walnut) [23], respectively. (**B**) Chromosomal localization position and duplication types of the *CBF* genes in *Juglans regia* (‘Chandler’) [20], *J. regia* (‘Zhongmucha-1’) [43], and *Juglans sigillata* (iron walnut) [23], respectively. The genome information of the three walnuts is shown at the bottom of each figure of chromosomal locations. The red dot and green dot indicate Tandem duplication and dispersed duplication.

The chromosomal distribution of *CBF* and *ICE1* genes was determined using the genome annotation data(*.gff). For the three cultivars (*Juglans regia*), the *CBF* distribution was different. The *CBF* genes of ‘Chandler’ were located on four distinct chromosomal: Chr01, Chr02, Chr03, and Chr10. Only one *CBF* gene was located on each chromosome (Figure 2A). For ‘Zhongmucha-1’, there were seven *CBF* genes distributed on six chromosomes, with two *CBF* genes on Chr3 (*JreCBF3* and *JreCBF6*, Figure 2A). In addition, the five *CBF* genes from ‘Yangbi’ walnut were dispersed on five different chromosomes (Figure 2A). According to the *ICE1* distribution analysis, there were two fewer members in ‘Yangbi’ walnut than the two other walnut types (six *ICE1* genes in ‘chandler’ and four in ‘Zhongmucha-1’, respectively; Figure 2B). Two *JrICE1* genes were located on Chr01 and Chr10, separately (Figure 2B).

To further explain the complexity and diversification of *CBFs* and *ICE1s*, we conducted a duplication mode analysis (Table S2). A total of three duplication patterns in *Juglans regia* (‘Chandler’, ‘Zhongmucha-1’, and ‘Yangbi’) were observed in which the main mode was whole-genome duplication (WGD) event, which occurred at the maximum proportion. In total, 15 out of 16 *CBFs* (93.75%) and 10 out of 12 *ICE1s* (83.33%) experienced the WGD event. Otherwise, only *JreCBF6* exhibited tandem duplication (TD), and two *ICE1* genes (*JrICE1c* and *JrICE1c*) experienced dispersed duplication (DSD) (Figure 2; Table S2). The result of the duplication analysis indicated that WGD induced the expansion of the CBF and ICE1 gene family in *Juglans regia* (Figure 2; Table S2).

### 2.3. Protein Domain and Gene Structure Analysis

**Figure 3:**
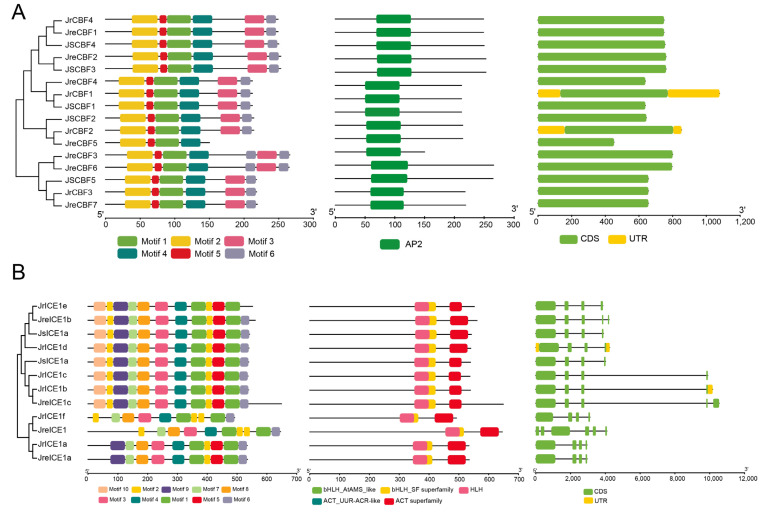
Motifs, protein domains, and gene structures of *CBF* (**A**) and *ICE1* (**B**) in *Juglans regia* (‘Chandler’) [20], *J. regia* (‘Zhongmucha-1’) [43], and *J. sigillata* (‘Yangbi’) [23], respectively. The Neighbor-Joining (NJ) phylogenetic tree of two genes in three *Juglans* assemblies is shown on the left. The motifs and protein domains are represented by different colored boxes. Green boxes and gray lines indicated exons and introns, respectively.

To exhibit the protein domain, motifs, and gene structure of *CBF* and *ICE1* with respect to similarity, the phylogenetic tree (Neighbor-Joining tree) was devised based on the protein sequences (Figure 3). The results showed that all CBF proteins contained only one conserved domain named AP2, and ICE proteins had five conserved domains (named bHLH_AtAMS_like, bHLH_SF superfamily, HLH, ACT_UUR-ACR-like, and ACT superfamily, respectively). The five domains overlapped in the ICE protein of bHLH_AtAMS_like and bHLH_SF superfamily, HLH, ACT_UUR-ACR-like, and ACT superfamily (Figure 3B). The motifs of the CBF proteins were similar in terms of size and type (six motifs) but were different in terms of locations (Figure 3A). The same methods were found for the ICE protein, including similar motifs; however, some ICEs had variations, such as JrICE1 that lacked motif9 and JrICE1e that lacked motif6, respectively (Figure 3B). For gene structures, overall, the *CBF* genes generally had one CDS of long length of one mainly CDS (~600 bp) in three walnut genomes, while there were at least four CDSs (one longer CDS and other shorter CDSs) in walnut *ICE* genes (Figure 3). In addition, *JrCBF1* and *JrCBF2* had UTRs of long length, indicating that they play regulatory functions. Although the ICEs had conserved motifs and domains, three *ICE* genes (*JrICE1c*, *JrICE1b*, *JreICE1c*) contained introns of long length (Figure 3). 

### 2.4. Collinearity Analysis and Selective Pressure

**Figure 4:**
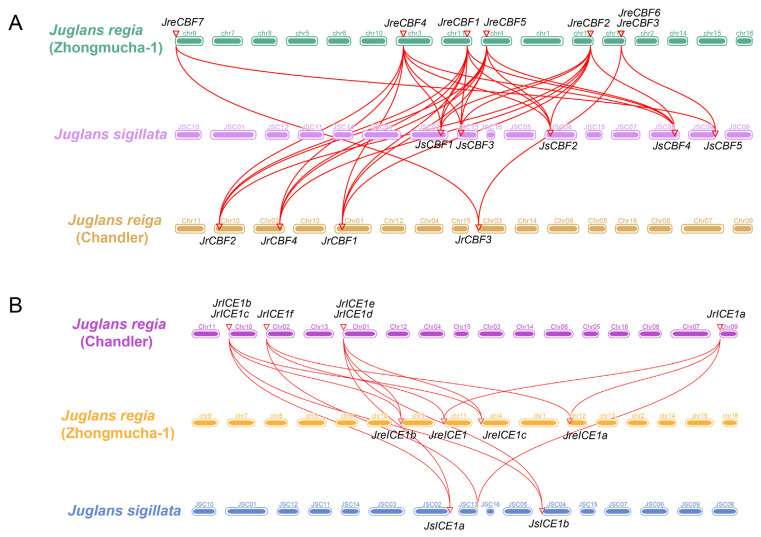
Genome-wide collinearity analysis for *CBF* (**A**) and *ICE1* (**B**) among *Juglans regia* (‘Chandler’) [20], *J. regia* (‘Zhongmucha-1’) [43], and *J. sigillata* (iron walnut) [23], respectively. Red lines indicate orthologous gene pairs.

Among the walnut genomes, all *CBF* and *ICE* genes had collinearity relationships (Figure 4). The collinearity predictions showed that there were eight *CBF* and eight *ICE* paralogous gene pairs and 44 *CBF* and 26 *ICE* orthologous gene pairs between the three *Juglans* genomes, respectively (Figure 4 and Figure S1). Among these genes, four *CBFs* (*JreCBF4*, *JreCBF1*, *JreCBF5*, and *JreCBF2*) had higher collinearity with other *CBF* genes (Figure 4 and Figure S1A). For *ICEs*, the analysis showed that each *ICE* gene had at least two collinearity relationships with other *ICEs*, suggesting that all *ICE* genes were very similar and conserved. Both *JsICE1a* and *JsICEb* had three collations with *ICE* genes of *J. regia* (Figure S1B). Although there were different members of *CBF* and *ICE* groups in the three walnut genomes, these two gene families were highly correlated (Figure 4). To further reveal the selection pressure of the homologous gene pairs of *CBFs* and *ICEs* in the three walnut genomes, we determined the Ka/Ks values of these homologous *CBF* and *ICE* gene pairs (Table S3). The Ka/Ks ratios of eight homologous gene pairs were more than 1, while the other CBF and ICE homologous gene pairs were less than 1 (Table S3). The Ka/Ks value was more than 1 for eight orthologous gene pairs (*JsCBF1* vs. *JreCBF5*, *JrCBF1* vs. *JsCBF1*, *JrCBF1* vs. *JreCBF4*, *JrICE1b* vs. *JsICE1a*, *JsICE1a* vs. *JreICE1c*, *JrCBF2* vs. *JsCBF2*, *JrICE1b* vs. *JreICE1b*, and *JrICE1f* vs. *JrICE1a*), suggesting that they were occurred positively selections and may be experienced a rapidly evolutionary rate. Within the eight gene pairs, only one in the same genome (*JrICE1f* and *JrICE1a*). In addition, the Ka/Ks ratios were less than 1, indicating that these gene pairs experienced relatively purify selection during evolution (Table S3).

### 2.5. Cis-Acting Elements Analysis of Promoters

**Figure 5:**
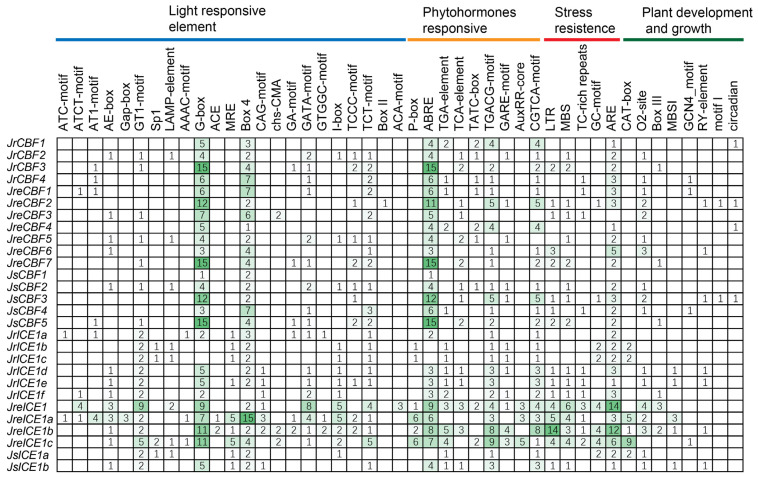
*Cis*-acting element analysis in promoter regions of *CBF* and *ICE1* genes in two to three walnut genomes. The *cis*-acting elements are shown on the top of the functional annotations, and they are divided into four major categories: light responsiveness *cis*-acting elements, phytohormone responsiveness, stress resistance, and plant development and growth. The gene names are provided on the left, and the number of *cis*-acting elements is given in the colored green box.

To investigate the potential functions of the *CBF* and *ICE* genes in the three walnut genome assemblies, we conducted a *cis*-acting element analysis of promoter regions of these two genes. The *cis*-acting elements were divided into four major classes, namely those with a light-responsive element, phytohormones responsive, stress resistance, and those associated with plant development and growth (Figure 5). The upstream promoter regions of the main *CBF* and *ICE* genes contained *cis*-acting elements associated with stress resistance, indicating that these *CBF* and *ICE* genes may play an essential role in light, abiotic stress, and resistance in the walnuts. In addition, the promoter CBF regions of the ‘Zhongmucha-1’ walnut genome contained a larger number of cis-acting elements than those of CBFs in the other two walnut genomes (‘Chandler’ and ‘Yangbi’), suggesting that *CBFs* in walnuts may be involved in genetic variations in the cold stress signaling and pathways. We also found that the ICEs of three walnuts contained a large number of *cis*-acting elements in the promoter regions of LTR and ARE related to stress resistance. Furthermore, most of the *CBF* and *ICE* genes contained the *cis*-acting elements associated with three light responsive, namely, G-box and box4, while namely the ABRE (related to the abscisic acid hormone response), CGTCA-motif and TGACG-motif (related to the methyl jasmonate hormone response) genes associated with phytohormones response were found in all three walnut varieties (Figure 5). 

### 2.6. Gene Expression Patterns Analysis

**Figure 6:**
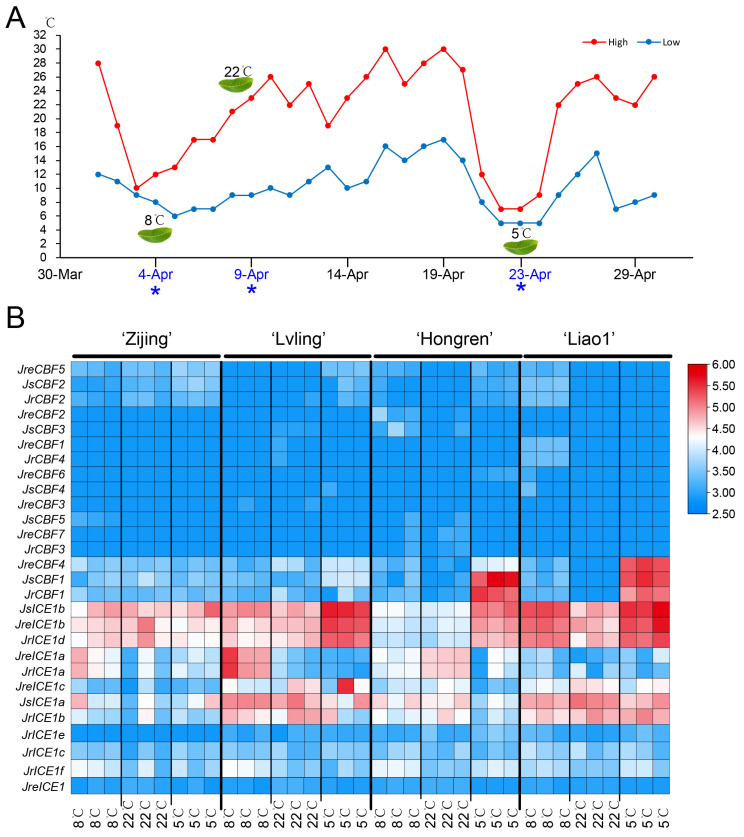
The graph of high and low temperature each day in April at Xi’an (**A**) and expression patterns of *CBF* and *ICE1* genes of three walnuts in four walnut cultivars, ‘Zijing’, ‘Lvling’, ‘Hongren’, and ‘Liao1’, under three temperature conditions (4 °C, 8 °C, and 21 °C), respectively (**B**). The blue symbol * and blue words indicate that plant materials fresh leaves from four cultivars under three low temperatures: 8 °C (4 April), 22 °C (9 April), and 5 °C (23 April), respectively.

To investigate the *CBF* and *ICE* expression patterns in four walnut cultivars (‘Zijing’, ‘Lvling’, ‘Hongren’, and ‘Liao1’) under different temperature conditions, transcriptomic data from leaves under three levels of temperature (8 °C, 22 °C, and 5 °C) were analyzed (Figure 6 and Figure S3; Tables S4–S6). Based on the transcriptome data, we evaluated the DEGs (differentially expressed genes) between the three different temperature conditions (8 °C, 22 °C, and 5 °C) in the four walnut cultivars (Figure S3; Table S4). A total of 18,740 DEGs were identified between the three different temperature conditions, including 465 shared DEGs in three comparison pairs (Figure S4; Table S5). Some of the annotated genes relating to plant growth, development, and stress in those overlapped in 465 DEGs (Table S5), such as four Auxilin-like protein (AUL), two AP2/ERF, three Ethylene-responsive transcription factor (ERF4), three Gibberellin 2-beta-dioxygenase (GA2OX), five E3 ubiquitin-protein ligase (ATL), two F-box protein, four Calcium-binding protein (CML), two Caffeoyl-CoA O-methyltransferase (CCOAOMT), five Cytochrome P450 (CYP), one Dehydrin (COR), four NAC domain-containing protein (NAC), Transcription factor bHLH96, six transcription factor (MYB), four WRKY transcription factor (WRKY), three Omega-3 fatty acid desaturase (FAD), and four Pentatricopeptide repeat-containing protein (PCMP).

We performed the statistical analysis of the different expression levels of *CBF* genes and *ICE* genes under three different temperature conditions (8 °C, 22 °C, and 5 °C) among four walnut cultivars (Figure S5). The results showed that *CBF* genes were expressed relatively significantly differently between the walnut cultivars at 22 °C and 5 °C (Figure S5A), while there were no significant differences in the *ICE* genes (Figure S5B). In addition, we found that the *ICE* genes were expressed significantly higher than *CBF* genes under the three different temperature conditions (8 °C, 22 °C, and 5 °C) in the four walnut cultivars (Figure S5C). Similarly, the linear relative regression analysis of the expression levels of the four cultivars was different for the CBFs and ICEs (Figure S5D). We performed an analysis of variance (ANOVA) for the three temperatures and four cultivars. The results showed that there was a significant difference in the expression level among the three temperatures (*p* = 2.7 × 10^−8^ ***), among the four cultivars (*p* = 0.027 *), and between the temperature and cultivars (*p* = 0.00045 ***), respectively. For the *CBF* gene, there was a significantly different expression level among the four cultivars (*p* = 0.00289 **), but not among the temperatures or between the temperature and cultivars (Table S7). In addition, the ANOVA of the *CBF* and *ICE* genes showed that 14 of 28 (50%) were expressed significantly differently among the three temperatures, among the four cultivars, and between the temperature and cultivars, including *JreCBF1*, *JreCBF2*, *JreCBF4*, *JreCBF5*, *JrCBF1*, *JrCBF2*, *JrCBF4*, *JsCBF1*, *JsCBF2*, *JreICE1a*, *JreICE1b*, *JrICE1a*, *JrICE1d*, and *JsICE1b* (Table S8). Furthermore, we found that only one of sixteen *CBF* genes (6.25%) was expressed significantly at 5 °C for the walnut cultivars ‘Hongren’ and ‘Liao1’, but the *CBF* gene was expressed slightly less at 5 °C for the cultivars of ‘Zijing’ and ‘Lvling’ (Figure 6B). We only found three *ICE* genes that were expressed to a lesser degree in all of the walnut samples, while three *ICE1* genes (*JsICE1b*, *JrICE1b*, and *JreICE1d*) were overexpressed at 5 °C for all four cultivars (Figure 6 and Figure S5). Based on the three sets of ANOVA results, for the temperatures, there were seven genes (*JreCBF4*, *JreCBF5*, *JreCBF6*, *JrCBF2*, *JrCBF4*, *JsCBF1*, and *JrICE1e*) were expressed significantly differently among the four cultivars at 8 °C, four genes (*JreCBF1*, *JrCBF1*, *JsCBF2*, and *JreICE1e*) at 22 °C, and three genes (*JrCBF2*, *JsCBF2*, and *JreICE1e*) at 5 °C, respectively (Table S8). For the cultivars, there were seven genes (*JreCBF1*, *JreCBF4*, *JreCBF5*, *JrCBF1*, *JrCBF2*, *JsCBF1*, and *JsCBF2*) in ‘Liao1’ were expressed significantly differently under the three temperature conditions, three DEGs (*JreCBF5*, *JsCBF3*, and *JreICE1a*) in ‘Lvling’, three DEGs (*JrCBF1*, *JsCBF1*, and *JreICE1*) in ‘Hongren’, and one DEG (*JsCBF5*) in ‘Zijing’ under the three temperature conditions (Table S9). In general, the *ICE* gene expression was significantly higher than for *CBF* genes (Figure S5C). All of the statistical analyses of gene expression suggested that most *ICE* genes regulated the expression in walnut leaves after the temperature decreased, while *CBF1* might be the most important transcription factor in walnut in terms of cold stress. Both *CBF* and *ICE* genes had different expression profiles in the four walnut cultivars under the three temperature conditions in the same environment, suggesting that different walnut varieties may have different cold stress resistance mechanisms. 

### 2.7. Protein–Protein and Protein-microRNA Interaction Predictions 

**Figure 7:**
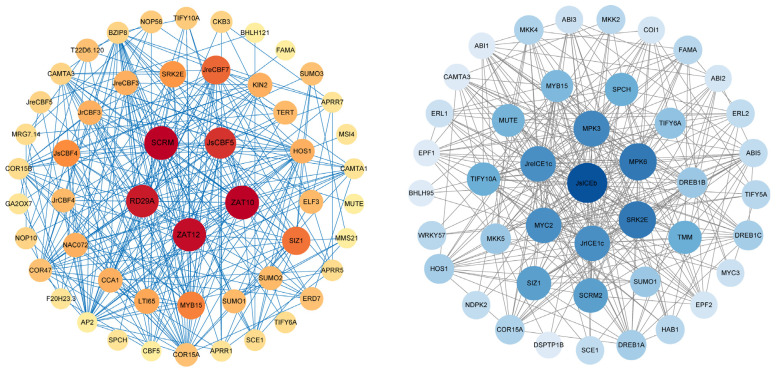
Protein–protein interaction networks of CBF and ICE with other proteins. The color line indicates protein interactions. The darker color indicate that the more numbers of protein-protein interaction relationships.

We investigated the CBF and ICE proteins and other proteins interactions, and the results showed that a total of seven CBFs and three ICEs, including JsCBF5, JrCBF4, JsCBF4, JrCBF3, JreCBF3, JreCBF5, JreCBF7, JsICE1b, JreICE1c, and JrICE1c, interacted with other proteins, such as SRK2E (SUCROSE NONFERMENTING 1-RELATED PROTEIN KINASE), ZAT12 (ZINC TRANSPORTER OF ARABIDOPSIS THALIANA), ZAT10, RD29A (RESPONSIVE TO DESICCATION 29A), MYB15 (MYB DOMAIN PROTEIN), MPK3 (MITOGEN-ACTIVATED PROTEIN KINASE), MPK6, MYC2, WRKY57, ABI2 (ABA INSENSITIVE), and ABI5 (Figure 7). These protein–protein interactions suggest that CBF and ICE play key functions related to cold-stress proteins, such as MYB15 and WRKY57. In addition, we found that both CBF and ICE proteins interacted with overlapped proteins, including SRK2E, FAMA (Encodes a basic helix-loop-helix transcription factor), MUTE (Encodes a basic helix-loop-helix transcription factor), CAMTA3 (Encodes a putative CAM binding transcription factor), COR15A, TIFY6A (TIFY DOMAIN PROTEIN), and HOS1 (HIGH EXPRESSION OF OSMOTICALLY RESPONSIVE GENES) (Figure 7). Furthermore, JsCBF5 and JsICE1b had more protein interaction relationships compared to other CBF and ICE proteins. 

To better understand the CBF and ICE protein interactions, we further performed the statistical and annotation analysis of the interaction proteins (Figure 7; Table S10). A total of 40 and 37 proteins exhibited interactions with CBF and ICE, respectively. There were 11 overlapping interaction proteins with CBF and ICE proteins, including CAMTA3, FAMA, HOS1, MYB15, SCE1 (SUMO CONJUGATING ENZYME), SIZ1 (SALT INDUCED ZINC FINGER PROTEIN1), SPCH (SPEECHLESS encodes a basic helix-loop-helix transcription factor), SRK2E, SUMO1 (Encodes a small ubiquitin-like modifier), TIFY10A, and TIFY10A (Figure 7; Table S10). The HOS1 was a negative regulator of cold responses, and other proteins were related to stresses such as MYB15, which was a key regulator of lignin biosynthesis in effector-triggered immunity; SRK2E, which might be involved in the ABA signaling network; and TIFY10A, which is associated with the response to a jasmonate stimulus. Among the unique interaction proteins in CBFs, there were 7 of 29 (24.1%) relative to cold response, including AP2, bZIP8 (basic leucine-zipper 8), COR15B, KIN2 (COLD-RESPONSIVE), RD29A, SCRM (SCREAM), and ZAT12 (Table S10). Within the 26 unique interaction proteins in CBFs, there were 16 (61.5%) relative to cold response and the ABA signaling network, including MKK4 (mitogen-activated map kinase), EPF2 (Encodes a secretory peptide), MPK3, MPK6, ABI1, ABI2, ABI3, ABI5, DREB1A, DREB1B, DREB1C, HAB1 (HYPERSENSITIVE TO ABA), DSPTP1B (DUAL-SPECIFICITY PROTEIN PHOSPHATASE), SCRM2, and ZAT12 (Figure 7; Table S10).

**Figure 8:**
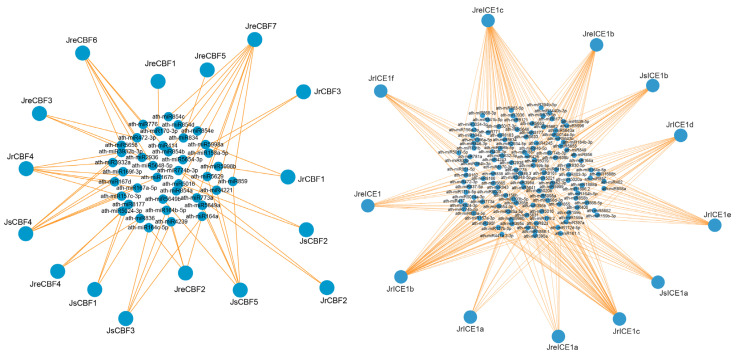
Representation of the regulatory networks between the targeted CBF (**left**) and ICE (**right**) genes and putative miRNAs. The bigger circles represent proteins, and the small circles represent miRNAs. The colored line indicates interaction relationships.

In plants, miRNAs play many biological functions by regulating the expression of target genes, such as in clod tolerance [44,45,46]. There is no published miRNA database in psRNATarget for walnut species, so we predicted the interaction network between the CBF and ICE proteins in walnuts and the putative miRNA in Arabidopsis for target prediction. All *CBFs* and *ICEs* had at least one interaction with miRNA in our analysis. The *ICE* genes had more putative miRNA interactions (124) compared to *CBF* (39 miRNAs) in walnuts (Figure 8; Table S11). For the *CBF* genes, *JreCBF7* had the highest number of nine putative miRNA interactions, while *JrCBF1* had only one putative miRNA (ath-miR170-3p) interaction. A total of 12 *CBF* genes had at least three miRNA interactions. For the *ICE* genes, *JrICE1c* had the highest number of putative miRNA interactions at 47, while *JrICE1a* had 9 putative miRNA interactions. All 12 *ICE* genes had at least 9 miRNA interactions with an average interaction of 23 (Figure 8). On the other hand, ath-miR164b-5p and ath-miR5021 targeted the most *CBF* genes (*JreCBF2*, *JsCBF3*, *JrCBF4*, and *JsCBF4*) and *ICE* genes (*JrICE1d*, *JrICE1e*, *JrICE1b*, *JrICE1c*, *JreICE1b*, *JreICE1c*, *JsICE1a*, and *JsICE1b*), respectively. In addition, *JrICE1b* interacted with a total of 45 miRNAs, and it expressed highly in leaves under three temperature conditions. In addition, there were 14 shared interaction miRNAs between the *CBF* and *ICE* genes, including ath-miR2936, ath-miR164a, ath-miR164b-5p, ath-miR164c-5p, ath-miR472-3p, ath-miR5024-3p, ath-miR773a, ath-miR5648-5p, ath-miR8177, ath-miR5654-3p, ath-miR5998a, ath-miR5998b, ath-miR774b-3p, and ath-miR5016 (Table S11). These miRNAs are mostly related to salinity, drought, and cold stress (Table S11). 

### 2.8. Evolution of CBF1 and ICE1 in Angiosperms 

**Figure 9:**
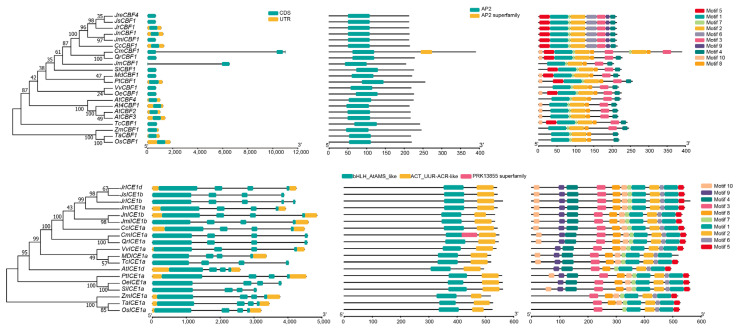
Phylogenetic analysis, gene structure, conserved domains, and motif of CBF1 and ICE1 in 19 angiosperms. The numbers on the NJ tree indicate that the bootstrap rate.

The *CBF1* and *ICE1* genes have been identified as major regulatory TFs of cold-related proteins in many angiosperm plants [5,6,7,14,15]. To better understand the evolution and variations of CBF1 and ICE1 proteins in the walnut family and other plants, we selected a total of 19 plant genomes, including six *Juglans* varieties (*J. regia*, ‘Chandler’; *J. regia*, ‘Zhongmucha-1’; *J. sigillata*, ‘Yangbi’; *J. nigra*, *J. microcarpa*, and *J. mandshurica*), three monocotyledons (*Oryza sativa*, *Zea mays*, and *Triticum aestivum*), and another ten plants (*Carya cathayensis*, *Castanea mollissima*, *Quercus robur*, *Sesamum indicum*, *Malus pumila*, *Populus trichocarpa*, *Vitis vinifera*, *Olea europaea*, *Arabidopsis thaliana*, and *Theobroma cacao*). The phylogenetic tree of CBF1 and ICE1 showed that the species have converged into the same group with close systematic relationships; for instance, all of the CBFs and ICEs of *Juglans* are clustered into one group, except for *JmCBF1* (Figure 9). Three CBFs and three ICEs of the monocotyledons were clustered into one group of more than 85, all CBFs and ICEs of Arabidopsis were clustered into a single group, and the CBF1 and ICE1proteins of Fagales were also clustered into one group in the phylogenetic tree. These results suggest the convergent evolution of CBF1 and ICE1 in angiosperms, which is also supported by the conserved domains in these two proteins. The CBF1 proteins had only one conserved domain, named AP2, while the ICE1 proteins had two conserved domains, named bHLH_AtAMS_like and ACT_UUR-ACR-like, in all nineteen plant genomes. The same patterns of gene structure and motifs were found for CBF1 and ICE1, which suggests that all CBF1 and ICE1 had conserved protein domains, motifs, and structures. However, CmCBF1 had one more domain, named AP2 superfamily, and CmICE1 had one more domain, named PRK13855 superfamily, respectively. In addition, we found that CmCBF1 and JmCBF1 had long introns between two CDSs, which suggests that there is some genetic variation among the *CBF* genes in plants. In our study, we found some long UTRs in many ICEs from the 19 plant species (Figure 9). The ICE genes were long in length, and the UTRs differed between species. The UTRs were also differently located among the plant species. This phenomenon was also observed in the *CBF* genes, such as *CcCBF1* and *OsCBF1* had long UTRs. Furthermore, we found that one long CDS in all *ICE1* genes and at similar locations in all of the plant species, while shorter CDSs were distributed differently among the species (Figure 9).

## 3. Discussion

The C-repeat binding factors (CBFs) and ICE transcription factors are well known to play essential roles in plant development, growth, and stress responses, particularly in regulating cold and chilling responses in plants [1,2,3,47]. They are important candidate genes for abiotic stress, such as in the cold acclimation processes [47], while 12 CBFs were found to be responsive to low temperature (4 °C) in lettuce [44], *LpCBFs* were expressed significantly under cold treatment conditions in *Lolium perenne* [48], and GthICE2 has been shown to respond to cold and drought stress [18]. Gene function experiments have been carried out in model plants; such as *AtCBF1* has been found to be involved in response to low temperatures and abscisic acid in *A. thaliana* [49,50], *OsCBF3* is responsible for low-temperatures-induced expression in rice [51], and *SlCBF1* enhances the resistance to cold tolerance and water deficit stress in tomato (*Solanum lycopersicum*) and *Brassica napus* [52]. Thus, a genome-wide investigation of the CBF and ICE gene family will be useful to elucidate plant cold stress. However, there is little information available about the CBF and ICE gene family information in the economic perennial tree, walnut. In this study, the two most important two TFs gene families of CBF and ICE were identified and analyzed in three walnut genomes using bioinformatic methods, including the examination of phylogenetic trees, gene chromosome locations, gene structure, motifs, conserved domains, expression levels, and protein interaction networks, as well as evolutionary relationships.

### 3.1. Characteristics of CBF and ICE1 in Three Walnut Genomes

Walnut is an important food nut and oil tree species that is widely cultivated across the world for its valuable nuts and wood [20,21,22]. It is cultivated extensively for nut and wood production [53]. *J. regia* is widely grown in diverse temperature regions of Asia, Europe, America, Australia, South Africa, and New Zealand [53]. As its sister taxon, *J. sigillata* (iron walnut), is only grown in the southwest of China, these two walnuts are growing sympatric and exhibit strong gene introgressions [23,24,25]. A rapid change in the climate can damage the buds, flowers, young stems, and even the entire plant [31,32,33]. Climate change also affects the growth of tree species in their distribution region, and in particular, low temperatures affect plant development and growth [23,30]. The transcription factors CBF and ICE have evolved into an important aspect of functional genomics research; thus, the genome-wide identification and gene functional research of the essential TFs in woody plants becomes increasingly more significant for genome sequencing and technology development [20,21,22,23,24,25]. Here, we identified a total of 16 CBFs and 12 ICEs in three walnut genomes. All putative *CBF* genes were divided into three groups, while the *ICE* genes were clustered into four groups. The number of *CBF* and *ICE* genes varied in the three walnut varieties, indicating the diversity of these three walnut genomes. Among the *CBF* and *ICE* genes in the three walnuts, most of the genes (25/28, 89.3%) exhibited whole genome duplication events (WGDs), while *JrCBF6* exhibited tandem duplication and *JrICE1e* and *JreICE1* showed dispersed duplication. The members of the CBF and ICE gene families are associated with the size of the genome and the effects of plant evolution [28]. This is in contrast to the number of CBF family members in annual plants; for example, there are 4 in *A. thaliana*, 12 in *Lolium perenne* [48], 14 in lettuce [47], 32 in the biennial herb *Brassica oleracea* [54], and 24 in the perennial herbs *Taraxacum kok* [55]. In the perennial woody plants, there are some reports of the presence of CBF members in whole genomes; for instance, in three *Acer* species, a total of five, four, and seven CBFs were identified in *Acer truncatum*, *Acer pseudosieboldianum*, and *Acer yangbiense*, respectively [8]. There were few reports of *ICE* genes in the genome-wide identification of plants. The use of domain query revealed that there were five, four, six, seven, eight, seven, eight, and eight genes coding the *ICE* domain in cotton (*Gossypium*) of *G. arboteum*, *G. raimondii*, *G. thurberi*, *G. hirsutum*, *G. barbadense*, *G. tomentosum*, *G. mustelinum*, and *G. darwinii*, respectively [10]. Differences in the number of CBFs and ICEs among the angiosperms can be explained by gene duplication, genetic diversity, and evolution. Differences in the molecular weight (16.68–90.91 kDa), number of amino acids (150–648), and isoelectric point (4.98–10.00) of walnut CBFs and ICEs indicate putative differences in the walnut genomes. All CBF and ICE TFs are primarily localized to the nucleus according to the subcellular localization prediction analysis, and similar results have been reported in other plants [8,9,10]. 

### 3.2. Conserved Domain and Cis-Acting Elements of CBF and ICE1 in Three Walnut Genomes

The previous studies showed that the conserved domains in transcription factors (TFs) might be related to gene functions, and the *cis*-acting elements play roles in the regulation of genes and post-transcriptional modification; similar results have also been found in other plant studies [28,37,39]. All CBF proteins contained only one domain, AP2, indicating that they play key roles in plant growth, development, and stress, which is consistent with the previous studies [54,55]. The AP2 domain is presented specifically in plants but not animals and important TFs belong to the superfamily AP2/ERF [28,56,57,58,59]. We found the consistent and conserved CBF proteins in three walnut genomes, which have also been found in other plants [9,10,11,12]. The similar pattern and conserved domains of CBF and ICE proteins in different plant species suggest that it is useful for CBFs and ICEs to be identified as diverse in terms of plant evolution and environmental adaption, which further demonstrates the essential nature of these two families for plant growth, development, and stresses response [16,17,18,60]. Although the identified CBF and ICE protein domains and motifs were conserved, their gene structure was different in the three walnut genomes, particularly ICEs (Figure 2). The *CBF* genes were structurally conserved in Arabidopsis and other plants (with one conserved exon), and the length and sequence of the CDS were also highly conserved, while a few species had two exons, such as *Castanea mollissima* and *Juglans mandshurica* [28]. For the *ICE* genes, there was high diversity in the gene structure in *Juglans* species and other plants (Figure 9). In the present study, we found that the number of exons in many ICEs of the 19 plant species was greater than 3, and all members had long exons (Figure 9). The ICE genes had long lengths and conserved exons between species, and the exons were also differently located among the plant species, which suggests that *ICE* genes play important roles and varieties during plant evolution and keep the conserved domains for their functions [61,62]. This phenomenon was not observed in the *CBF* genes of other plant species, so it might be unique to *ICE* genes. Furthermore, we found that the introns were very short in the *CBF1* genes but very long introns in the *ICE1* genes, which suggests that they have different benefits and regulation mechanisms in plants [63]. Both UTR and introns could lead to an increase in protein diversity and are involved in gene expressions [63] 

*Cis*-acting elements are well-known participants in the control of gene expression [37,38,48]. In our study, many *cis*-acting elements associated with responsiveness to light and phytohormones were identified in the upstream CBF and ICE promoter regions (Figure 4), which suggests that both CBF and ICE proteins might be associated with photosynthetic and hormone responses and regulations. As a light-preferring tree species, walnuts mainly grow in temperate forest areas [21,22,23]. Light is an important element that regulates plant growth, development, and stress. Many studies have shown that light can affect the cold stress in plants via *CBF* and *ICE* genes [2,3,4,5,6]. Plant hormones are well known to regulate plant stresses, and the hormones play very important roles in plant adaptions and growth in diverse environments [64,65]. Thus, these results revealed that CBF and ICE might play a critical role in the stress response of walnuts. In addition, we found some *cis*-acting elements associated with stress resistance, suggesting that *CBF* and *ICE* genes are also involved in some kinds of stress responses. 

### 3.3. Gene Expression Profiles of CBF and ICE1 in Response to Cold Stress

Currently, cold stress has become one of the essential factors limiting the flower and fruit productivity of walnuts, and the exploration of important salt and drought tolerance genes is key for the advancement of local walnut adaption breeding programs [1,2,3,20,27,28,29,30]. To reveal the *CBF* and *ICE* gene expression profiles, we detected the RNA sequencing data to calculate the FPKM values in different cold conditions and tissues. Most *CBF* and *ICE* genes (9 out of 10) were expressed more highly in the leaves compared to the flowers, hulls, and other tissues; however, five out of six *ICE* genes (*JrICE1a*, *JrICE1b*, *JrICE1c*, *JrICE1d*, and *JrICE1e*) were highly expressed in *J. regia* (‘Chandler’) female flowers, while all four CBF genes were less expressed in female flowers (Figure S2). Two *ICE* genes, *JrICE1d* and *JrICE1e*, were expressed highly in all tissues. These results indicated that both *CBF* and *ICE* genes play specific roles in different tissues and organs in walnuts. Among these *CBF* and *ICE* genes, *JreCBF4*, *JrCBF1*, *JsCBF1*, *JreICE1e*, *JsICE1a*, and *JrICE1b* were expressed highly in leaves at 5 °C after the temperature decreased quickly in two walnut cultivars, ‘Hongren’ and ‘Liao’, which suggests that both *CBF* and *ICE* played an important role during rapidly changes in the temperature in spring (Figure 6). In addition, four walnut varieties had different gene expression patterns of *CBF* genes and *ICE* genes under three temperature conditions (Figure 5 and Figure S5, Tables S6 and S9), suggesting that walnut species have experienced diverse environmental adaption through natural selection and domestication [20,21,22]. This is possibly related to the temperate climate of the natural habitat of iron walnuts in southwest China, mainly in Yunnan province [23,24]. Walnut species are distributed and cultivated in widely diverse ecologically regions in different climate areas in the world [20,21,22,25,26,27]. Previous reports showed that CBFs activate many downstream genes, and ICE1 acts upstream to regulate CBFs, which increases cold stress in plants [66]. Our analysis of *CBF* and *ICE* gene expression patterns under rapid temperature changes in the leaves of four walnut cultivars also suggested that *CBF* and *ICE* genes might play critical roles in the cold stress response of *J. regia* and *J. sigillata* (Figure 6), a finding that is also supported by those from other woody plant species [11,13,23]. Due to the potential role that the identified *CBF* genes and *ICE* genes play in walnut varieties with increased cold tolerance, CBFs and ICEs will be candidate genes to improve the resilience of walnut and woody crops to climate change.

### 3.4. Protein–Protein and Protein Interactions, and the Putative microRNA Interaction Predictions

In this study, we investigated the interactions between CBF and ICE proteins and other proteins (Figure 7). Our results showed that there were a total of eleven shared interaction proteins for both CBF and ICE proteins, including CAMTA3, FAMA, HOS1, MYB15, SCE1, SIZ1, SPCH, SRK2E, SUMO1, TIFY10A, and TIFY10A. The proteins had potential functions related to cold tolerance. The *Arabidopsis HOS-1* mutation regulated the genes *RD29A*, *COR47*, *COR15A*, *KIN1*, and *ADH* (*ALCOHOL DEHYDROGENASE*), and it mediated the FLC (FLOWERING LOCUS) and degradation of ICE1 as an important negative regulator of cold signal transduction in plant cells [67,68,69]. The important transcription factor MYB15 positively regulated cold stress by activating the gene expression of *CBFs* (*CBF1*, CBF2, and *CBF3*), targeted by Syl-miR156e-3p and ABA-mediation in tomato, but MYB15 interacts with the *ICE1* and negatively expression of *CBFs* in *Arabidopsis* [70,71,72,73]. In addition, *PUB25* (*PLANT U-BOX*) and *PUB26* negatively regulate MYB15 and positively regulate *CBF* expression in *Arabidopsis* [72,73]. SIZ1 is a small ubiquitin-related modifier (SUMO) E3 ligase that mediates ICE1 and CBF3 to enhance cold tolerance in *Arabidopsis* [74]. Within the unique interaction proteins in CBFs and ICEs, there were 7 and 16 proteins relative to cold response, respectively (Table S10). The AP2/ERF is an important transcript factor that positively enhances cold tolerance in plants, has been identified in *Juglans mandshurica* [28], and positively regulates cold stress in *Arabidopsis*, rice, and grape [75,76]. These shared and unique interactions among proteins will be useful resources and could be very important candidates in walnut cold tolerance studies. 

MicroRNAs (miRNAs) play an important role in regulating target gene expression and are key regulators in plant development and stress responses [45]. In woody species, some miRNAs have been identified, but little is known about these in walnuts. We found that a total of 14 miRNAs interacted with both CBFs and ICEs. Both ath-miR472-3p and ath-miR5024-3p have been reported to respond to cold tolerance by regulating their target genes in wheat and *Arabidopsis* [77,78]. A total of four miRNAs are related to salt, and four miRNAs are related to drought tolerance, suggesting that miRNAs play important roles in plant responses to abiotic stresses. Low temperature is a major environmental factor affecting the productivity of woody plants. These findings related to CBFs and ICEs interaction proteins and miRNAs indicate that protein–protein, and protein-microRNA interaction predictions could provide useful analysis and gene function studies in walnut cold tolerance breeding.

## 4. Materials and Methods

### 4.1. Plant Materials and RNA Sequencing

In this study, we used four cultivars of Persian walnut (*Juglans regia*), including ‘Zijing’, ‘Lvling’, ‘Hongren’, and ‘Liao1’, respectively. All trees were grown in Xi’an Botanical Garden, Shaanxi Province, China. We collected the leaf samples from four cultivars after a rapid decrease in the temperature in cold late spring. In April 2023, we collected the plant materials fresh leaves from four cultivars under three low temperatures: 8 °C (4 April), 22 °C (9 April), and 5 °C (23 April), respectively (Figure S5A; Table S2). We collected three biological duplications for each temperature condition and each cultivar. Then, the tissue samples were flash-frozen in liquid nitrogen and stored at −80 °C prior to utilization. The total RNA was extracted to analyze further the genes that responded to the rapid decrease in temperature. All thirty-six leaf samples were sequenced using the Illumina HiSeq X Ten platform (Illumina, San Diego, CA, USA). All of the transcriptome clean reads were then mapped to the *Juglans regia* (‘Chandler’) reference genome using HISAT version 2.2.0 [20,79] with default parameters. The numbers of fragments per kb of transcript sequence per million bp sequenced (FPKM) were used to calculate the gene expression levels [80]. We identified the DEGs (differentially expressed genes) between the three temperature conditions (8 °C, 22 °C, and 5 °C) and combined the four walnut cultivars (‘Zijing’, ‘Lvling’, ‘Hongren’, and ‘Liao1’) using DESeq2 (https://support.bioconductor.org/tag/DeSeq2/, accessed on 2 May 2023) [81,82] with fold change (FC) > 1 and FDR < 0.05. The volcano plot was employed to show the different expression patterns of upregulated and downregulated genes using the R package ggplot2 [83,84]. We analyzed the shared and unique DEGs between the three temperatures using the online program jvenn (https://jvenn.toulouse.inrae.fr/app/index.html, accessed on 2 December 2023) [85]. We further annotated the overlapped DEGs using the *Juglans regia* (‘Chandler’) reference genome [20].

### 4.2. Identification of the Gene Members in Three Juglans Regia Cultivars

To identify the candidate CBF and ICE transcription factors, homologous sequences of *Arabidopsis thaliana* (AT4G25470.1, AT4G25490.1, AT5G51990.1, AT1G12610.1, AT1G63030.1, AT1G63030.2) as the query target were downloaded from the TAIR database (https://www.arabidopsis.org/, accessed on 12 April 2023). We used three walnut chromosome-level genomes, *J. regia* (‘Chandler’) [20], *J. regia* (‘Zhongmucha-1’) [43], and *J. sigillata* (‘Yangbi’) [23], as the reference (e-value < 1 × 10^−50^, identity bits > 50) [35]. The CBF and ICE protein sequences of *A. thaliana* were adopted as query sequences to perform the genome-wide BLASTP for *J. regia* (‘Chandler’), *J. regia* (‘Zhongmucha-1’), and *J. sigillata* (‘Yangbi’), respectively [20,23,43]. We used the CBF and ICE Pfam numbers (PF00847 and PF00010) to build the HMM models and search in the whole-genome protein databases, and then we determined the candidate members of CBF and ICE combined with the HMMER search method using the parameters of scores > 50 and E-value > 10^−5^. The candidate genes contained the N-terminal motif (PKKP/RAGRxxKFxETPHP), and the DSAWR conserved domain sequence of the CBF protein could be the feature of the further screen. Similarly, the ICE1 protein contained distinctive conserved S-rich region motifs, a bHLH signature conserved domain, and a specific sequence containing 14 amino acids (KMDRASILGDAID/EYLKELL) at the N-terminus, and SUMO binding sites (IKEE/VKEE) and an NLS signal region at the C-terminus. We examined all of the CBF and ICE candidate protein sequences using the Pfam database (http://pfam.xfam.org/, accessed on 12 April 2023), the CDD (conserved domain database) (https://www.ncbi.nlm.nih.gov/Structure/cdd/cdd.shtml, accessed on 12 April 2023), and the SMART database (http://smart.omicstudio.cloud, accessed on 12 April 2023) used by the domain analysis program with an E value cutoff of 1.0 [86,87]. We used these conserved motifs and domains to verify the final gene members of CBF and ICE, respectively.

### 4.3. Construction of Phylogenetic Tree and Analysis of Gene Structure, Conserved Motif

The phylogenetic relationships among the *CBF* and *ICE* gene families were analyzed using the software MEGA version 7.0 using the Neighbor-Joining (NJ) tree with a bootstrap of 1000. Then, the Neighbor-Joining tree was embellished using the online tool iTOL (https://itol.embl.de/, accessed on 12 April 2023). Based on the CBF protein sequences belonging to *Arabidopsis thaliana*, *Juglans regia*, *J. nigra*, *J. mandshurica*, and *Cyclocarya paliurus*, we performed gene structure analysis using the website GSDS (http://gsds.gao-lab.org/, accessed on 12 July 2023). To visualize the candidate genes, the conserved motifs were predicted with a maximum of 10 motifs using the MEME program and the software TBtools version 2.008 [88]. The gene domain was explored using Batch CD-Search in NCBI (https://www.ncbi.nlm.nih.gov/Structure/bwrpsb/bwrpsb.cgi, accessed on 12 July 2023). Additionally, the detected domain was visualized using software TBtools version 2.008 [88]. 

### 4.4. Chromosomal Location, Collinearity of Transcription Factors, and Selective Pressure Analysis in Juglans Regia 

To identify the location of the genes on each chromosome, the target gene annotation information was retrieved from the *Juglans regia* whole genome annotation files. According to the physical position of the candidate *CBF* and *ICE* genes, the chromosomal locations of the *CBF* genes and *ICE* genes of *J. regia* (‘Chandler’), *J. regia* (‘Zhongmucha-1’) and *J. sigillata* (‘Yangbi’) were determined using the software TBtools version 2.008 [88]. The collinearity and duplication analyses were performed using McScanX software (https://github.com/wyp1125/MCScanx, accessed on 12 May 2023) [89]. All of the results above were visualized using the software TBtools version 2.008 [88]. In addition, we calculated the Ka/Ks values to estimate the selective pressure among the identified gene pairs using KAKS_CALCULATOR version 2.0 software [90]. The values of nonsynonymous (Ka), synonymous (Ks), and Ka/Ks were estimated in each CBF and ICE gene pair in three walnut genomes using the method of Yang and Nielsen (YN) [91]. Following the screening, several gene pairs were not listed if either the Ka or Ks values were unavailable for analysis. The Ka/Ks values more than 1 and less than 1 indicate positive selection and negative selection, respectively.

### 4.5. Analysis of Subcellular Localization and Protein Physicochemical Properties

The physicochemical properties of identified candidates were predicted by the online tool Expasy (https://www.expasy.org/, accessed on 13 July 2023). The subcellular localization of all genes was predicted using Protein Subcellular Localization Prediction: WoLF PSORT tools (https://wolfpsort.hgc.jp/, accessed on 12 July 2023).

### 4.6. Cis-Acting Elements Analysis of Promoter and Gene Expression

Based on the upstream 2000 bp sequences of the candidates extracted from the whole-genome files, the promoter sequences were uploaded to the online website PlantCARE database (http://bioinformatics.psb.ugent.be/webtools/plantcare/html/, accessed on 12 July 2023). For the transcriptome gene expression analysis, we first filtered all RNA raw sequencing data using the software FASTP (https://github.com/OpenGene/fastp, accessed on 1 May 2023) [92]. We utilized FASTP to ensure quality control and perform adapter trimming, quality filtering (limit the percentage of low-quality bases, default 40%), and per-read quality pruning, among other operations, to attain clean data from raw data for further analysis [92], and then the clean reads data were mapped to the reference genome of *J. regia* (‘Chandler’) using the software HISAT version 2.2.0 [79]. Then, the gene expression level was calculated using the FPKM values via the program featureCounts [84]. Subsequently, a heatmap was displayed using the software TBtools version 2.008 [88]. For the statistical analysis of the expression levels of CBFs and ICEs in the four walnut cultivars under three temperatures, we first performed the Bartlett test of expressions for these studied genes. Then, the ANOVA and Kruskal–Wallis test were employed to compare the differences amongst the different cultivar groups and temperature conditions, respectively. All statistical analysis was conducted using R version 4.2.3 software. The MANOVA (multivariate analysis of variance) and MRA (multiple regression analysis) were executed using R software to estimate the influence of temperature and cultivar on the gene expression level, respectively.

### 4.7. Protein–Protein Interactions and microRNA Targeting Analysis

We annotated all CBF and ICE protein sequences using the eggnog-mapper tool (http://eggnog-mapper.embl.de/ (accessed on 31 October 2023) [93]. The CBF and ICE protein sequences were uploaded to the STRING online website (https://cn.string-db.org, accessed on 31 October 2023) to analyze the protein–protein interaction predictions using default parameters [94]. Since there is no *Juglans* proteins–proteins and miRNAs interaction database, we mapped the representative CBF and ICE protein sequences with the *A. thaliana* genome using default parameters [93,95]. We analyzed the PPI prediction in STRING (https://cn.string-db.org, accessed on 31 October 2023) and performed the CBF and ICE genes with the putative miRNA interactions. We submitted all identified CBF and ICE proteins as candidates to predict the putative targeting of Arabidopsis miRNAs using the online program psRNATarget using default parameters [94]. Both protein–protein interactions and protein-microRNA interactions were visualized using the software Cytoscape version 3.10.0 with default parameters [96]. We analyzed the shared and unique proteins and miRNAs between the CBFs and ICEs using the online program jvenn (https://jvenn.toulouse.inrae.fr/app/index.html, accessed on 2 December 2023) [85]. We searched for the potential gene functions of all 14 overlapping interactive miRNAs on the NCBI website. We further annotated the overlapped proteins using the *Juglans regia* (‘Chandler’) reference genome [20].

### 4.8. Evolution of CBF1 and ICE1 Genes in Angiosperms

To further understand the important genes *CBF1* and *ICE1* genes in angiosperms, we used the one-to-one ortholog method to identify the two genes in a total of nineteen plant genomes (*J. regia*, ‘Chandler’; *J. regia*, ‘Zhongmucha-1’; *J. sigillata*, *J. nigra*, *J. microcarpa*, *J. mandshurica*, *Carya cathayensis*, *Castanea mollissima*, *Quercus robur*, *Sesamum indicum*, *Malus pumila*, *Populus trichocarpa*, *Vitis vinifera*, *Olea europaea*, *Arabidopsis thaliana*, *Theobroma cacao*, *Oryza sativa*, *Zea mays*, and *Triticum aestivum*,) (Table S1) [20,23,43,97,98,99,100,101,102,103,104,105,106,107,108,109,110,111,112,113]. We analyzed the phylogenetic relationships, conserved domains, gene structures, and motifs of all CBF and ICE proteins [88,95]. The neighbor-joining (NJ) phylogenetic trees of *CBF* and *ICE* genes were conducted using the software MEGA version 7.0 with a bootstrap of 1000. The conserved domains were analyzed using the Pfam, CDD, and SMART databases, respectively [86,87]. The conserved motifs were predicted with a maximum of 10 motifs using the MEME program in the software TBtools version 2.008 [88]. We performed the gene structure analysis of the identified CBFs and ICEs using the online website of GSDS (http://gsds.gao-lab.org/, accessed on 12 July 2023) and visualized using TBtools version 2.008 software [88].

## 5. Conclusions

In the present study, we identified a total of sixteen *CBF* and eleven *ICE* genes in three walnut genomes. The phylogenetic tree analysis showed that the *CBF* genes could be divided into three groups and that the *ICE* genes could be clustered into four groups, respectively. The phylogenetic, synchronic, and collinearity analyses indicated that both the *CBF* and *ICE* gene families were evolutionarily conservative. The CBFs had only one conserved domain, AP2, while the ICEs had bHLH and ACT conserved domains. Although the *CBF* genes were not evenly located across the chromosomes in the three walnut genomes, they exhibited consistently high collinearity, which revealed that the CBFs were relatively conserved proteins. Most of the *ICE* genes were distributed at the ends of the chromosomes in the three walnut genomes. The transcriptome expression profile analysis indicated that the *ICE* genes were expressed more highly than the *CBF* genes under cold conditions in four walnut cultivars (‘Zijing’, ‘Lvling’, ‘Hongren’, and ‘Liao1’), indicating that *CBF1* and *ICE1* have important functions under rapidly temperature changes in walnut leaves.

## Figures and Tables

**Table 1:** Information of CBF and ICE proteins from three walnut genomes.

Gene Name ^1^	No. of Amnio Acids	Mol. Wt (kDa)	Isoelectric Point (pI)	Instability Index (II)	Aliphatic Index	Grand Average of Hydropathicity (GRAVY)	Subcellular Localization
JrCBF1	212	23,251.1	5.26	52.72	61.37	−0.503	Nucleus
JrCBF2	214	23,955.9	6.20	56.34	66.17	−0.563	Nucleus
JrCBF3	218	24,001.2	8.59	54.02	71.24	−0.477	Nucleus
JrCBF4	249	27,626.1	5.53	57.99	72.13	−0.482	Nucleus
JreCBF1	249	27,626.1	5.53	57.99	72.13	−0.482	Nucleus
JreCBF2	253	28,821.2	5.42	56.81	65.61	−0.743	Nucleus
JreCBF3	266	29,507.2	6.19	55.73	70.49	−0.538	Nucleus
JreCBF4	212	23,251.1	5.26	52.72	61.37	−0.503	Nucleus
JreCBF5	150	16,684.8	10.00	59.52	60.60	−0.744	Nucleus
JreCBF6	265	29,260.9	6.19	60.5	68.94	−0.486	Nucleus
JreCBF7	218	24,001.2	8.59	54.02	71.24	−0.477	Nucleus
JsCBF1	212	23,251.1	5.26	52.72	61.37	−0.503	Nucleus
JsCBF2	214	24,026.0	6.75	54.69	62.52	−0.581	Nucleus
JsCBF3	253	28,893.2	5.33	56.32	65.61	−0.755	Nucleus
JsCBF4	250	27,799.2	5.21	56.64	70.28	−0.516	Nucleus
JsCBF5	218	24,011.3	8.59	54.9	71.24	−0.481	Nucleus
JrICE1a	534	57,836.5	5.36	46.26	74.01	−0.516	Nucleus
JrICE1b	539	58,329.4	5.03	56.61	78.89	−0.423	Nucleus
JrICE1c	537	58,102.2	4.98	56.64	78.99	−0.420	Nucleus
JrICE1d	540	58,754.7	5.16	56.23	72.46	−0. 542	Nucleus
JrICE1e	552	60,506.9	5.73	57.26	72.30	−0. 530	Nucleus
JrICE1f	492	54,205.0	5.77	46.78	77.36	−0. 515	Nucleus
JreICE1	645	70,822.6	6.35	49.98	83.32	−0. 305	Nucleus
JreICE1a	541	58,832.7	5.76	48.38	72.87	−0.563	Nucleus
JreICE1b	560	61,275.6	5.22	58.34	75.09	−0.521	Nucleus
JreICE1c	648	70,913.0	5.18	59.24	83.98	−0.330	Nucleus
JsICE1a	539	58,395.5	5.14	55.74	78.89	−0.441	Nucleus
JsICE1b	542	59,042.0	5.22	55.92	72.20	−0.550	Nucleus

^1^ The gene name of those CBF and ICE from three walnut genomes. Jr = *J. regia* (‘Chandler’) [20], Jre = *J. regia* (‘Zhongmucha-1’) [43], and Js = *J. sigillata* [23], respectively.

## Data Availability

Data is contained within this article and Supplementary Materials. The transcriptome data have been deposited in the NCBI under accession number: PRJNA1051792.

## Supplementary Materials

The following supporting information can be downloaded at: https://www.mdpi.com/article/10.3390/ijms25010025/s1.
