# Supplementary figures and images for: Genome-Wide Identification of the CBF Gene Family and ICE Transcription Factors in Walnuts and Expression Profiles under Cold Conditions
Source: Int J Mol Sci. 2023 Dec 19;25(1):25. doi: 10.3390/ijms25010025 (PMC10778614; doi:10.3390/ijms25010025)

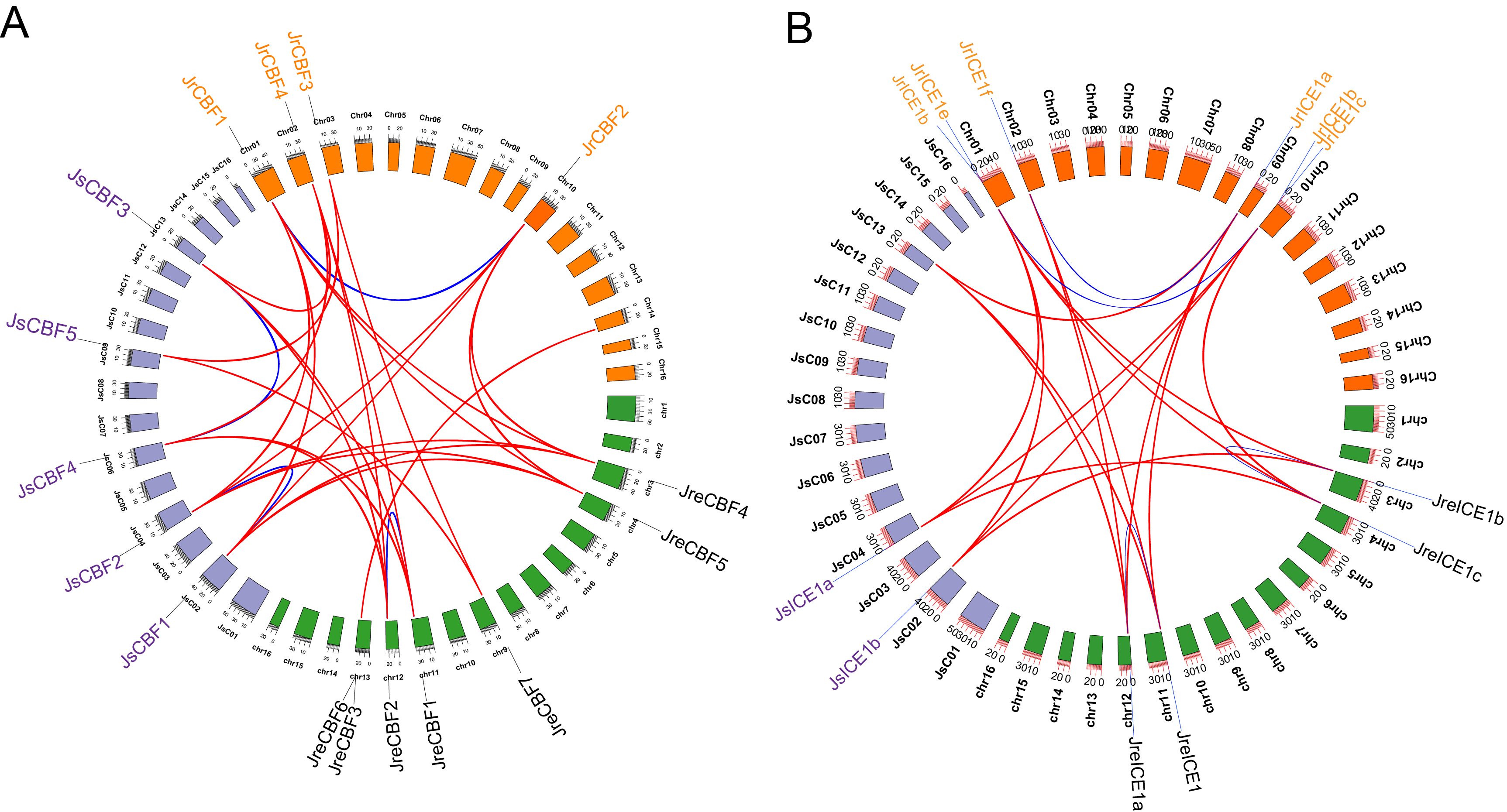

Supplement: Supplementary file 1 [file ijms-25-00025-s001.zip › Figure S1.tif]

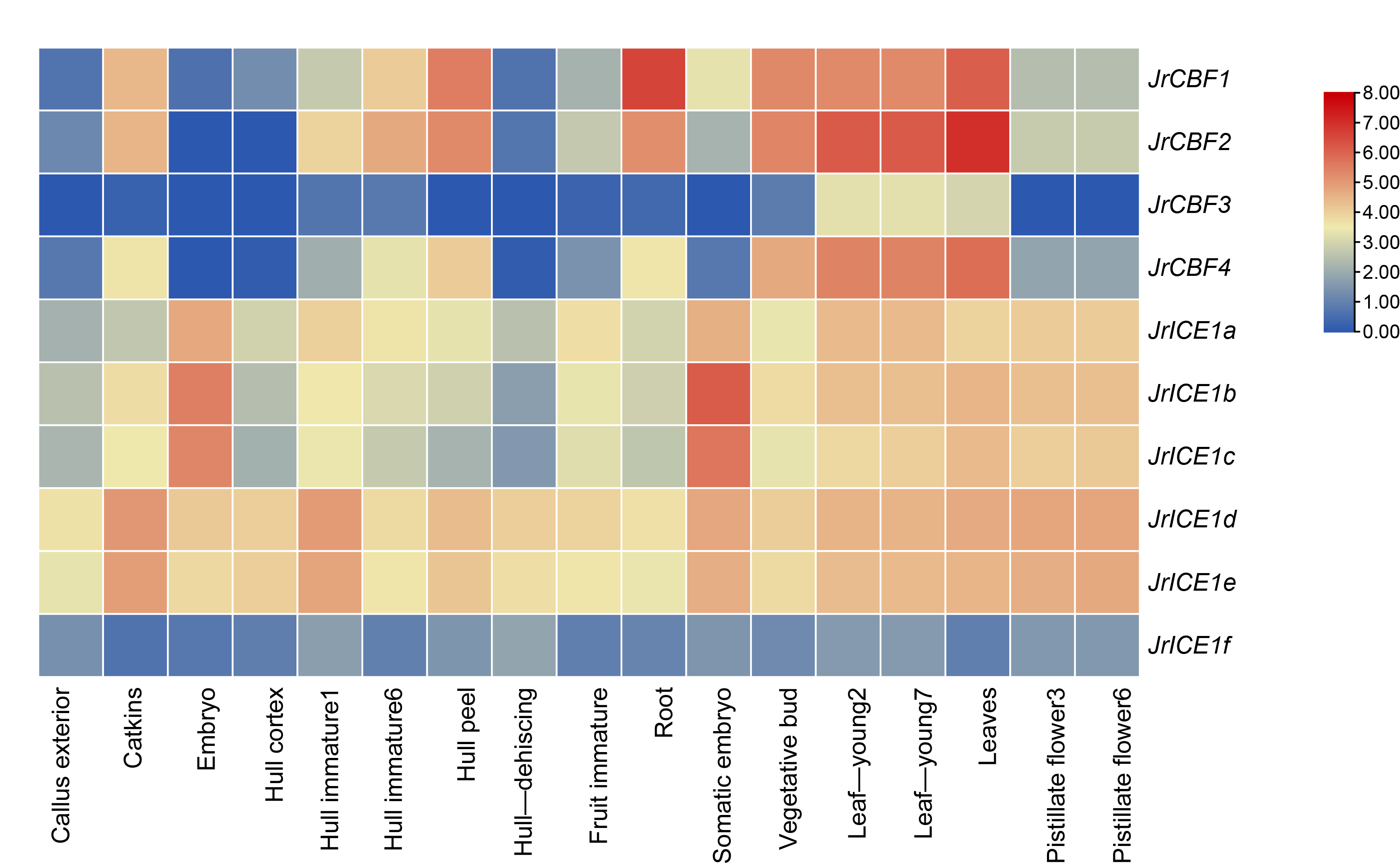

Supplement: Supplementary file 1 [file ijms-25-00025-s001.zip › Figure S2.tif]

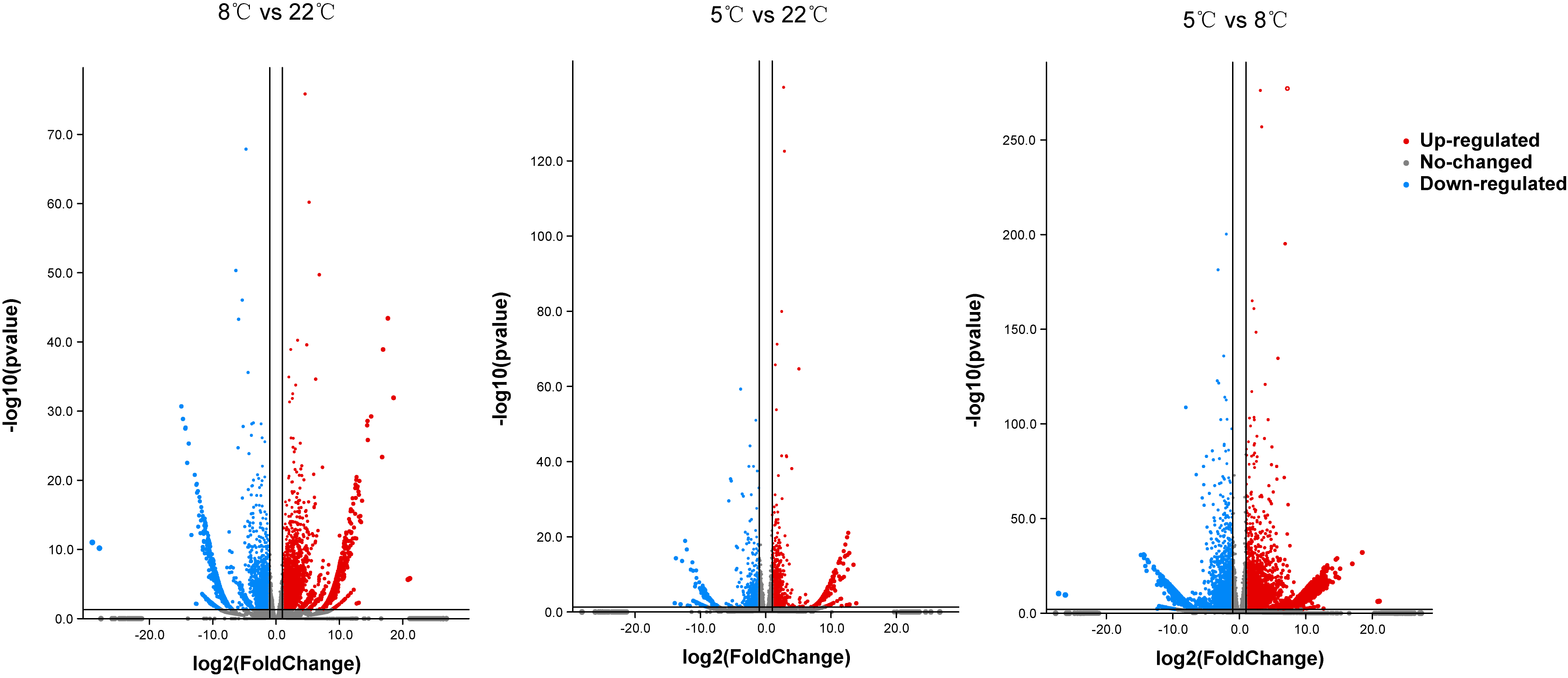

Supplement: Supplementary file 1 [file ijms-25-00025-s001.zip › Figure S3.tif]

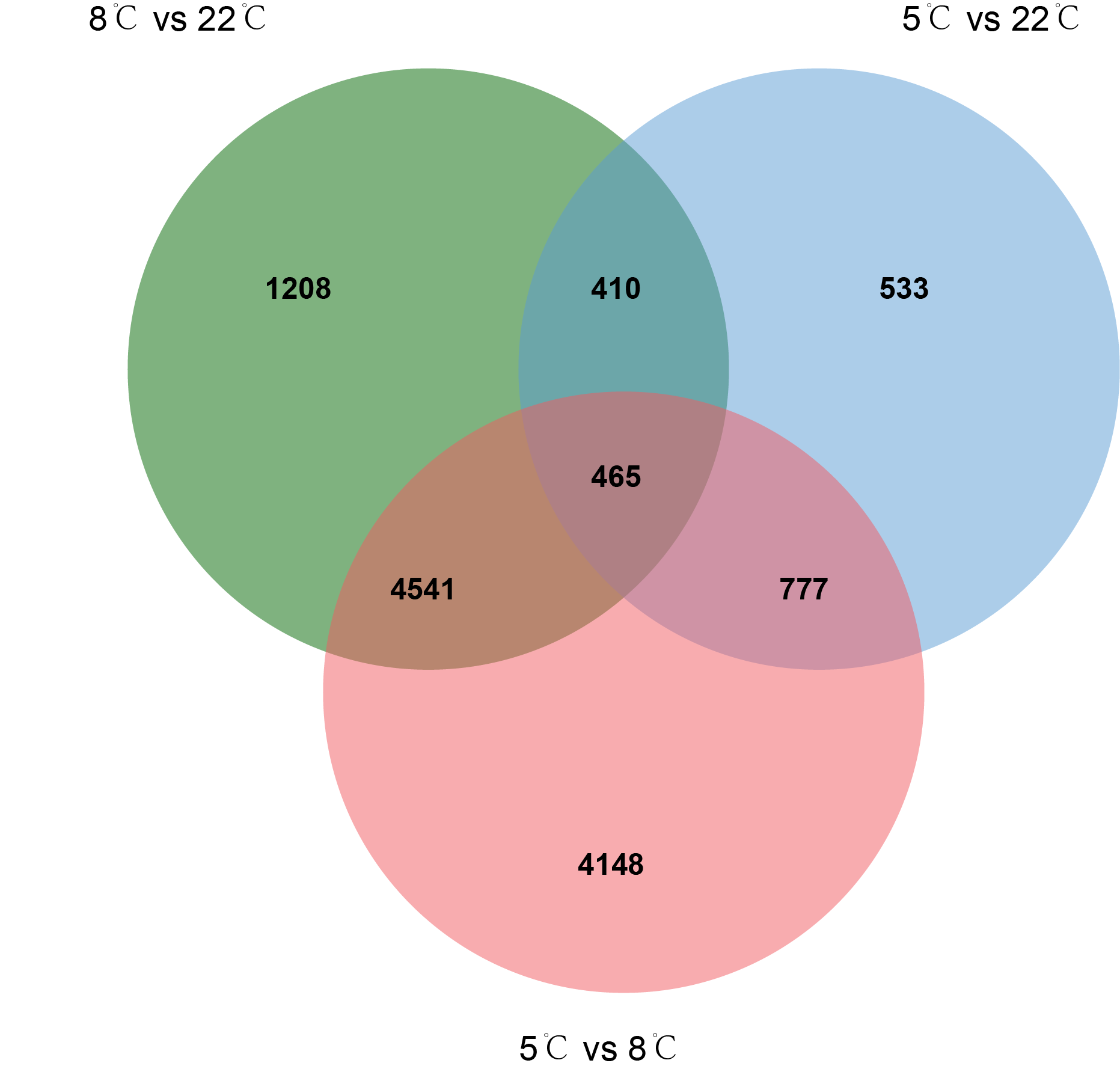

Supplement: Supplementary file 1 [file ijms-25-00025-s001.zip › Figure S4.tif]

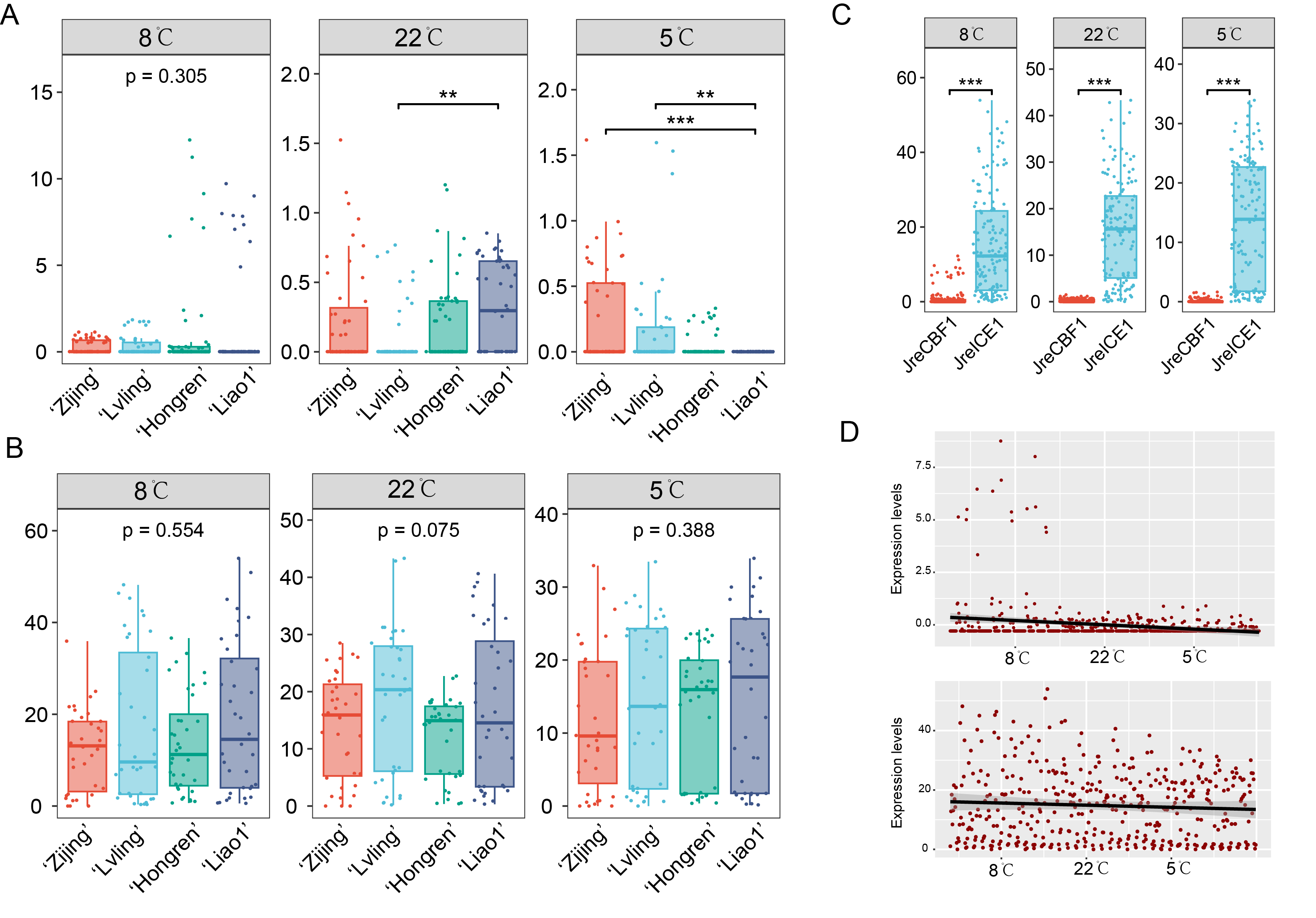

Supplement: Supplementary file 1 [file ijms-25-00025-s001.zip › Figure S5.tif]
